# Dominance of Carrying angle in Right-hand among Dental Students of a Teaching Hospital: A Descriptive Cross-sectional Study

**DOI:** 10.31729/jnma.7244

**Published:** 2022-03-31

**Authors:** Bipana Manandhar, Isha Shrestha, Ritee Shrestha

**Affiliations:** 1 Department of Anatomy, Kantipur Dental College, Basundhara, Kathmandu, Nepal; 2 Department of Physiology, Kantipur Dental College, Basundhara, Kathmandu, Nepal

**Keywords:** *elbow*, *hand*, *humerus*

## Abstract

**Introduction:**

Carrying angle is an acute angle formed between extended arm and forearm when palm is directed forward. This angle is formed due to the angulation of the articulating surfaces of the humerus with the forearm. The angle is greater in dominant hand than in non-dominant hand in both males and females. Thus, this study aims to measure the carrying angle and find the prevalence of dominance of the carrying angle in the right-hand among dental students.

**Methods:**

A descriptive cross-sectional study was conducted among 138 students with the age ranging from 18-23 years in a teaching hospital. Ethical clearance was taken from Institutional Review Committee (Reference number-28/021) of a tertiary dental college and teaching hospital. Convenience sampling was done. Carrying angle was measured in right and left-hands of students with the help of a goniometer. Statistical Package for the Social Sciences version 20.0 was used for data analysis. Point estimate at 95% Confidence Interval was calculated along with frequency and percentage for binary data.

**Results:**

Among 138 students, the dominance of carrying angle in right-hand was found in 107 (77.53%) (70.56-84.49 at 95% Confidence Interval). Prevalence of greater value of carrying angle in right-hand was found in 71 (78.88% ) female and 36 (75%) male students.

**Conclusions:**

The prevalence of dominance of the carrying angle in the right-hand among dental students was lower than the other studies done in similar settings.

## INTRODUCTION

Carrying angle of the elbow is an angle made by the long axis of the arm with the long axis of the forearm on extension.^1^ This angle is important in walking, swinging and carrying things. The angle is greater in the dominant hand than in the non-dominant hand in both males and females due to exert forces on the elbow joint. Greater carrying angle in dominant hand might be the influence of racial and growth factor.^2^ Medial flange of trochlea of humerus is six millimetres below the lateral flange leading to the formation of carrying angle.^3^ The average angle is about 5° to 10° in males and 10° to 15° in females.^4^

The knowledge of carrying angle is important in treatment of displayed elbow and management of elbow deformities and its reconstruction.

The main aim of this study was to find the prevalence of greater value of carrying angle in dominant right-hand among dental students in a teaching hospital.

## METHODS

This descriptive cross-sectional study was conducted in the Department of Anatomy, Kantipur Dental College and Teaching Hospital (KDCH), Basundhara, Kathmandu. The study was conducted from July 2021 to November 2021 after approval from the Institutional review committee (Reference number: 28/021) of KDCH. Purpose of study was well explained and informed consent was taken from the students. Students with no history of fracture and surgery of the upper limb bones were included in the study. Exclusion criteria included the students with dominant left-hand and endocrine disorders affecting skeletal systems. Convenience sampling was done and sample size was calculated by using the following formula:

n = (Z^2^ × p × q) / e^2^

  = (1.96^2^ × 0.5 × 0.5) / 0.09^2^

  = 119

Where,

n = required sample size
Z = 1.96 at 95% Confidence Interval (CI)
p = prevalence taken as 50%
e = margin of error, 9%

After adding a non response rate as 10%, the minimum required sample size was 132.

The study enrolled 138 students aged 18-23 years. They were made to stand in an anatomical position with their elbows extended. Tendon of Biceps brachii muscle at elbow & palmaris longus tendon at the wrist were palpated which marked the long axis of arm and forearm respectively. One arm of the goniometer was placed along the long axis of the arm and the other arm along the long axis of the forearm. Then, the carrying angle was recorded on the goniometer. Same method was repeated on the other arm also.

All the measurements were recorded and analysed statistically using Statistical Package for the Social Sciences 20.0 version software. Point estimate at 95% confidence interval was calculated along with frequency and percentage for binary data.

## RESULTS

Among 138 students, the dominance of carrying angle in right-hand was found in 107 (77.53%) (70.56-84.49 at 95% Confidence Interval). The present study was conducted among students of age ranging from 18-23 years. Right-hand dominance of carrying angle was more common in female students and observed in 71 (78.88%) among the total students who presented with carrying angle dominant in right hand (Table 1).

**Table 1:** Prevalence of dominance of carrying angle in right hand of students (n= 107).

Sex	Right-hand n (%)
Male	36 (75)
Female	71 (78.88)

Mean carrying angle in the right hand of total students was 12.04±1.61 (11.71-12.36 at 95% Confidence Interval). Mean value of carrying angle in the right hand of female students was found to be 12.58±1.60 which was slightly higher than the value of carrying angle 11.22±1.25 observed in males. The greatest value of carrying angle was measured to be 12.99° in the right hand of females and the least value was recorded as 10.82° in males (Table 2).

**Table 2:** Distribution of carrying angle among two different genders (n= 107).

Carrying angle	Sex	Mean±SD (degrees) (Range)
Right-hand	Male	11.22±1.25 (10.82-11.62)
	Female	12.58±1.60 (12.16-12.99)

## DISCUSSION

Knowledge of dominance of carrying angle in the right hand is useful in treating elbow disorders and injuries for reconstruction. This study revealed the dominance of carrying angle in right-hand with 77.53%. We observed a higher value of carrying angle in right-hand in 71 (78.88%) female students and 36 (75%) male students. This study showed a higher mean value of carrying angle (12.58±1.60) in the right-hand of female students whereas the value was a little lower (11.22±1.25) in the right-hand of male students.

Right-hand dominance of the carrying angle of this study (77.53%) was lower than the right-hand dominance (93.9%) of a study conducted among volunteers of southeastern Nigeria.^5^ Right-hand dominance was observed in 69.82% of students and employees of Jordan University of Science and Technology, Jordan.^6^ Our study showed right-hand dominance (77.53%) slightly higher than this study. Right-hand dominance of this study was found lower than the right-hand dominance (96.5%) of the study conducted among healthy volunteers of South Indian origin.^7^

Our study was similar to the study conducted among medical students of a medical college of India which revealed the greater carrying angle in the right-hand and carrying angle of females was found more than that of males.^8^ This study resembled the study conducted among students of a medical college of Nepal in which the mean carrying angle was higher in the right-hand.^9^ Our study was consistent with the study conducted among individuals from Nigeria which resulted with greater value of carrying angle in right-hand (9.75 + 2.260) of females which was more than that of males.^10^ This could be due to hormonal differences in males and females and waist circumference of females could be the cause for the higher mean value of carrying angle in females than in males. In a study conducted among male and female patients at Department of Orthopaedics and Traumatology of UNIFES,^11^ they found elbow carrying angle to be 12.88±5.92° in males and 15.07±4.95° in females. Our findings coincide with their findings as females presented with greater carrying angles than males.

This study was consistent with the study conducted among young adult participants which presented the mean carrying angle in the right-hand of males as 9.77±2.82°and13.94±3.97° i n females.^12^ The findings of our study supported the findings of the research done on MBBS students of Bidar Institute of Medical Sciences Bidar, Karnataka, India^13^ in which higher value of carrying angle was observed in right-hand and females having carrying angle higher than males.

Our study was in contrast with the study conducted among students of Kathmandu University High School, Chaukot^14^ in which the value of carrying angle was found to be greater in the left hand. But the similarity of our study with this study was the finding of greater value of carrying angle in female students than in male students. Our study did not coincide with the study conducted among Bangladeshi Garo people^15^ which revealed higher carrying angle in males than in females.

Carrying angle helps to keep our hand in position with stability.^16^ Increased carrying angle may cause pain in different movements of hand.^17^ Development of right and left-hand can occur asymmetrically which could be the cause for greater prevalence of carrying angle in right-hand of students. Prevalence of carrying angle found more on the right-hand of females than males could be the influence of their genetic background and hormonal difference. Racial and age factors may affect the value of carrying angle. Greater carrying angle in females may be due to laxity in ligaments of elbow joint. Broader hips compared to males could be the factor leading to greater carrying angle in females than in males.

The limitations of this study was that the variation of carrying angle with age and ethnicity has not been studied. Interobserver variation might influence the readings taken on the goniometer. Further studies in a larger population with an equal number of males and females would be ideal and recommended.

## CONCLUSIONS

The prevalence of dominance of carrying angle in the right hand of students was found lower than the other studies done in similar settings. The knowledge of greater value of carrying angle in right hand can be useful in the management of elbow disorders and treatment of its injuries. It will be also helpful for designing elbow prosthesis.
